# Socio-economic factors explain differences in public health-related variables among women in Bangladesh: A cross-sectional study

**DOI:** 10.1186/1471-2458-8-254

**Published:** 2008-07-23

**Authors:** Md Mobarak H Khan, Alexander Kraemer

**Affiliations:** 1 Department of Public Health Medicine, School of Public Health, University of Bielefeld, Germany

## Abstract

**Background:**

Worldwide one billion people are living in slum communities and experts projected that this number would double by 2030. Slum populations, which are increasing at an alarming rate in Bangladesh mainly due to rural-urban migration, are often neglected and characterized by poverty, poor housing, overcrowding, poor environment, and high prevalence of communicable diseases. Unfortunately, comparisons between women living in slums and those not living in slums are very limited in Bangladesh. The objectives of the study were to examine the association of living in slums (dichotomized as slum versus non-slum) with selected public health-related variables among women, first without adjusting for the influence of other factors and then in the presence of socio-economic variables.

**Methods:**

Secondary data was used in this study. 120 women living in slums (as cases) and 480 age-matched women living in other areas (as controls) were extracted from the Bangladesh Demographic and Health Survey 2004. Many socio-economic and demographic variables were analysed. SPSS was used to perform simple as well as multiple analyses. P-values based on t-test and Wald test were also reported to show the significance level.

**Results:**

Unadjusted results indicated that a significantly higher percent of women living in slums came from country side, had a poorer status by household characteristics, had less access to mass media, and had less education than women not living in slums. Mean BMI, knowledge of AIDS indicated by ever heard about AIDS, knowledge of avoiding AIDS by condom use, receiving adequate antenatal visits (4 or more) during the last pregnancy, and safe delivery practices assisted by skilled sources were significantly lower among women living in slums than those women living in other areas. However, all the unadjusted significant associations with the variable slum were greatly attenuated and became insignificant (expect safe delivery practices) when some socio-economic variables namely childhood place of residence, a composite variable of household characteristics, a composite variable of mass media access, and education were inserted into the multiple regression models. Taken together, childhood place of residence, the composite variable of mass media access, and education were the strongest predictors for the health related outcomes.

**Conclusion:**

Reporting unadjusted findings of public health variables in women from slums versus non-slums can be misleading due to confounding factors. Our findings suggest that an association of childhood place of residence, mass media access and public health education should be considered before making any inference based on slum versus non-slum comparisons.

## Background

People living in slums and informal settlements are growing rapidly all over the world especially in the developing countries [1]. Worldwide at least 1 billion people are estimated to live in slum communities [2] and experts projected that the number will rise to 1.6 billion by the year 2020 [1] and to 2 billion by 2030, whereas the global population is expected to increase from 6 to 8 billion by 2030 [3].

Slums are the spatial manifestations of urban poverty, social exclusion, and inappropriate government policies [3] and often characterized by one or more of these shortcomings: deteriorated or poorly structured houses crowded together, insecurity of tenure, poor environmental managements such as deficient access to safe drinking water and sanitation, stagnation of water and poor drainage with excessive open sewers, excessive amount of uncollected rubbish, severe overcrowding, flies, and poor lighting [4-6]. These settings are also dominated by a migratory population living under stressful conditions. Evidences from Bangladesh indicate that migrants initially experienced housing shortages and usually settled in highly congested areas [7]. About two-third of the slum housing was constructed of low quality flimsy materials. A majority of the migrants (about 60%) lived in rental houses [7], did not meet the daily nutritional requirement [8] and were involved with informal occupations or low paying formal positions [4,8].

In Bangladesh, the slum population is also increasing at an alarming rate especially in the urban areas due to migration by the rural poor. For instance, the proportion of the population living in the slums of Dhaka city has increased from 20% in 1996 to 37% in 2005 [4]. Such a rapid growth of slum populations in Bangladesh is an increasing challenge for local health authorities and deserves intensive investigations [4,9]. Slums have often been conceptualized as areas of concentrated poverty [4], which comprise a social cluster that engenders a distinct set of health problems. This neglected population has become a major reservoir for a wide spectrum of adverse health conditions [2] such as undernutrition [8,10,11], delivery-related complications [12], postpartum morbidity [11,12], diabetes [13], fever [14], intestinal problems [14], measles [14], skin diseases [14], respiratory infections [14], pain [15], sexually transmitted infections [16], and high rates of smoking [17]. Diarrhoeal diseases [5,18], less vaccination coverage [9,19], malnutrition [20], chronic intrauterine under-nourishment [21,22], low birth weight [21,23], premature birth [21], high infant mortality rate [24,25], serious behavioural problems and post-traumatic stress disorders [26] are also more likely to occur in slum areas.

Poverty, poor housing, high population densities and inadequate living conditions combined with environmental conditions favoring vector breeding readily promote the spread of communicable diseases in poor communities [27]. Generally, overcrowding make poor residents vulnerable to contracting communicable diseases such as tuberculosis and acute respiratory infections. Transmission of these illnesses is often aided by low resistance among the population owing to malnutrition. Vaccine-preventable diseases such as measles, diphtheria, and whooping cough also spread more rapidly in overcrowded urban areas among non-immunized populations. Inadequate provision for drainage and sanitation raises the risk of malaria, dengue, and yellow fever [5]. Continued neglect of ever-expanding slum populations in the world could inevitably lead to a greater expenditure and diversion of health care resources to the management of diseases that are preventable [2].

Considering this worldwide phenomena, in the year 2000 the United Nations Millennium Declaration pledged to tackle the challenge of setting specific goals of achieving a significant improvement in the lives of at least 100 million slum dwellers by the year 2020 [2]. The Government of Bangladesh is also committed to achieve the targets embodied in the Millennium Declaration by 2015 [28]. To capture the real picture of people in urban slums, the National Institute of Population Research and Training (NIPORT), a governmental organization of Bangladesh, has conducted a large survey in six major cities called "Urban Health Survey" and obtained information about demography, living conditions, health, life-styles and so on (data not yet published). Although various studies regarding slum populations are available in Bangladesh, these are mostly confined to the slums of Dhaka city. Moreover, only few studies compared slum and non-slum populations focusing on certain aspects such as low birth weight [23], immunization coverage [19,29], rheumatic diseases [15], psychiatric disorders [26], smoking [17], and mental health and quality of life [30]. To our knowledge, none of these studies compared women living in slums with those living in other areas in the same vicinity/cluster. Hence, the objectives of the study were to examine the association of the variable slum (dichotomized as slum versus non-slum) with selected public health variables among women, first without adjusting for the influence of other factors and then in the presence of some socio-economic variables. Body mass index (BMI), knowledge of AIDS indicated by a question "ever heard about AIDS", knowledge of AIDS prevention indicated by condom use, antenatal visits, and safe delivery practices assisted by skilled sources were the public health related variables in this study.

## Methods

### Data sources

We used secondary data which was extracted from the Bangladesh Demographic and Health Survey (BDHS) conducted in 2004 [31]. This survey was nationally representative and carried out by a Bangladeshi research firm 'Mitra and Associates' under the authority of the National Institute for Population Research and Training (NIPORT) of the Ministry of Health and Family Welfare. Technical assistance was provided by ORC Macro through the MEASURE DHS program. Financial support for the survey was provided by the US Agency for International Development (USAID)/Bangladesh. The survey is intended to serve as a source of population and health data for policy makers and the research community in the country. Four different questionnaires shortly entitled as household, women, men and community questionnaire were used. The contents of these questionnaires were based on MEASURE DHS+ model questionnaire. These model questionnaires were adapted for use in Bangladesh during a series of meetings with the members of Technical Task Force, consisting of experts from both national and international organizations. Draft questionnaires were then reviewed by the BDHS Technical Review Committee.

### Duration of study

According to the national report of BDHS 2004 [31], the whole study period ranged from September 2003 to June 2004. For instance, pretesting of the women questionnaire was done in September 2003. A household listing operation was performed during October 3 to December 15, 2003. The data collection period through questionnaire extended from January 1 to May 25, 2004. Data processing such as coding, editing and entering was completed during January 12 to June 24, 2004.

### Selection of clusters and households

The BDHS 2004 included 361 clusters (enumeration areas) from both urban and rural areas based on the 2001 Bangladesh Population Census (BPC). For that census, enumeration areas were created based on a convenient number of dwelling units in both rural and urban areas. As sketch maps of enumeration areas were accessible, these were considered suitable to be used as primary sampling units for the BDHS 2004. This survey used stratified cluster sampling based on urban and rural areas. Initially 361 primary sampling units, 122 from urban areas and 239 from rural areas were selected. From each cluster, an average of 30 households was selected systematically and information through a household questionnaire survey was collected. A total of 10,811 households were then selected from 361 clusters.

### Household survey

The purposes of the household survey were to list all the members and visitors in the selected households. Some basic information was collected such as age, sex, education, marital status, and relationship to the head of the household. One of the main purposes of the household survey was to identify the eligible women and men for an individual interview. In addition, information was collected about the dwelling itself, such as whether the household was located in a slum or not, source of water, type of toilets, materials used to construct the house, and ownership of various consumer goods. In this survey 10,500 households were successfully interviewed (slum = 112, non-slum = 10,388; urban = 3,513 and rural = 6,897) from 10.811 (overall response rate = 99.8%) which provided a total of 51,255 persons. Each household member was indicated by a unique identification number consisting of three characteristics: cluster number, household number in the selected cluster and line number in the selected household. All the eligible women and men were selected from this household list for detailed women and men surveys later. The following section provides only information about the women survey.

### Women survey

For the women questionnaire survey, all the eligible women (criteria: ever married and aged 10–49 years) from the list of household survey were selected. The women questionnaire was used to collect information about many topics including background characteristics (such as age, education, religion, etc), anthropometric measures (e.g. height and weight), access to mass media, reproductive history, use of family planning methods (e.g. condom), awareness of AIDS and other sexually transmitted diseases, and antenatal and safe delivery practices. A total of 11,601 eligible women were identified from the household lists, of which 11,440 women (response rate = 98.6%) were successfully interviewed. Before starting interview, a verbal consent was obtained from each respondent by explaining the objectives of the survey. It was also assured that the information will be kept confidential. A higher number of eligible women (= 11,601) from 10,500 households indicated that some households had more than 1 eligible woman. In such a case, every woman was labelled by a different line number in the household. Every eligible woman bore the same identification number in both household and women surveys. These identification numbers were used to link two data sets collected by household and women surveys (see below). Such a linkage was necessary because the women questionnaire did not include the question whether the woman was living in a slum or not. In contrast, the household survey did not collect detailed information for eligible women.

### Linking of *household and women *data sets

Using the unique identification number, we linked two data sets and identified the women living in slums. Linkage data provided a total of 120 women living in 112 slum households from 11,440 women. At the next step, we identified 480 non-slum women (4 non-slum women per 1 slum woman) who were mainly matched by age living in the same or nearest cluster. The following steps were used for selection of the study sample:

Step 1: All the household members (such as male, all children <10 years old and women >49 years old) were deleted from the household data set to obtain a smaller data set.

**Figure 1:**
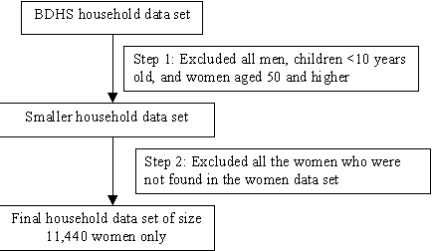
Reduction of household data set to match with women data set.

Step 2: Two data sets were matched by using identification numbers. If any woman of a household data set with a specific identification number was not available in the women data set (due to e.g. non-response), she was excluded from the household data. In this way the household data set was finally reduced to a size of 11,440 only, equal to the size of the women data set. The reduction was necessary because the household data set contained not only eligible women but also other members such as men and children. Figure 1 shows the first two steps mentioned above.

Step 3: We copied the slum variable (whether the woman was living in a slum household or not) from the final household data set and then pasted it into the women data set. In total, there were 120 women (cases) who were living in 112 households located in slums.

Step 4: We selected only those clusters (54 out of 361) which contained at least one woman living in a slum household. Other clusters were deleted from the data set, which reduced the sample of women from 11,440 to 1,777. According to the data, slum households were located in both urban and rural areas, with a higher rate in urban areas (e.g. 71 households in urban areas compared with 41 in rural areas although the total households were almost double in rural areas).

Step 5: For each individual woman living in slum, we selected 4 women living in non-slum who were mainly matched by ± 5 years of age and living in the same or nearest cluster. Only in some cases (clusters with higher number of women in a slum), we selected the non-slum women from the nearest clusters (based on geographical location) because the number of non-slum women in the same cluster was not sufficient for matching. In this way, we selected 480 women (controls) in non-slums to compare with 120 women in slums from both urban and rural areas. We selected the non-slum women from the same or nearest cluster in order to minimize some of the differences that may occur due to e.g. variation in environment, infrastructure, and health facilities by different clusters. According to the study design of BDHS 2004, it was unlikely that any women was interviewed twice in different places (e.g. in both urban and rural areas) during the survey.

### Independent variables/composite variables

Several socio-demographic variables were analyzed to explain the differences between women living in slums and non-slums (Table 1). Two composite variables, namely a composite variable of household characteristics and a composite variable of mass media access were also constructed by simply adding six and three individual dichotomous variables (shown below) respectively. Composite variables were used to capture more information by a smaller number of variables.

**Table 1:** Socio-demographic and mass media variables for women living in slums compared to those not living in slums in Bangladesh

Variables	Non-slum	Slum	p^¶^
	
	n (%)	n (%)	
Age in years (mean ± SD)	480 (30.1 ± 9.0)	120 (30.1 ± 9.6)	0.969
Age at first marriage (mean ± SD)	480 (15.7 ± 3.5)	120 (15.0 ± 3.2)	0.057
Number of living children (mean ± SD)	480 (2.4 ± 1.8)	120 (2.5 ± 1.9)	0.492
Number of children died (% at least one)	480 (24.4)	120 (30.0)	0.206
Education (% no education)	480 (40.8)	120 (31.3)	0.003
No. of marital union (% 2+)	480 (4.6)	120 (6.7)	0.349
Migrated from other place (% country side)	480 (69.4)	120 (81.7)	0.007
Have marriage certificate (% yes)	480 (79.6)	120 (70.8)	0.039
Currently working (% yes)	480 (20.6)	120 (25.8)	0.215
Husband's education (% no education)	480 (27.9)	120 (44.2)	<0.001
Husband's occupation (% unskilled labour)	479 (16.5)	120 (30.0)	0.013
**Household characteristics:**			
Floor material (% cement/concrete)	477 (43.0)	120 (15.8)	<0.001
Wall material (% brick/cement)	478 (43.7)	120 (18.3)	<0.001
Roof material (% cement/concrete)	478 (27.6)	120 (7.5)	<0.001
Piped water (% inside dwelling)	478 (27.4)	120 (13.3)	0.001
Toilet facility (% modern/septic tank)	478 (68.8)	120 (43.3)	<0.001
Cooking fuel (% gas/LPG)	478 (29.4)	120 (15.8)	0.003
*Composite variable of household characteristics (% not poor when > 0)*	*477 (76.5)*	*120 (52.5)*	*<0.001*
**Mass media variables:**			
Reading newspaper (% not at all)	480 (72.3)	120 (85.0)	0.004
Listening to radio (% not at all)	480 (51.9)	120 (56.7)	0.347
Watching TV (% not at all)	479 (27.6)	120 (35.0)	0.108
*Composite variable of mass media access (% not at all)*	*479 (17.5)*	*120 (25.0)*	*0.063*

^¶^t-test for continuous variables and χ^2 ^test for categorical variables

### Construction of composite variables

The composite variable of household characteristics was made by adding 6 household characteristics:

• Floor material (cement/concrete = 1, else = 0)

• Wall material (brick/cement = 1, else = 0)

• Roof material (cement = 1, else = 0)

• Piped water (inside dwelling = 1, else = 0)

• Toilet facility (modern/septic = 1, else = 0)

• Cooking fuel (Gas/Liquefied Petroleum Gas (LPG) = 1, else = 0)

After summing up, the total score for 6 variables varied from 0 to 6. If the total score was 0, then the composite variable of household characteristics was considered as "poor", otherwise it was considered as "not-poor".

Similarly, the composite variable of mass media access was determined by adding three variables:

• Read newspaper not at all (yes = 1, no = 0)

• Listened to radio not at all (yes = 1, no = 0)

• Watched TV not at all (yes = 1, no = 0)

The total score for the composite variable of mass media access varied from 0 to 3, where total score '0' indicated that the woman had not at all access to mass media". Score from 1 to 3 indicated that she had access to at least one mass media.

### Dependent variables

Five selected dependent variables namely (i) BMI (kg/m^2^), (ii) ever heard about AIDS, (iii) use of condom to avoid AIDS, (iv) number of antenatal visits during the last pregnancy, and (v) safe delivery practices assisted by skilled sources (composite variable, see below) were used for detailed analyses. One of the main justifications of choosing these variables was that they differed significantly by a simple (unadjusted) analysis when compared by slum and non-slum women. BMI was selected because it is widely used as an indicator of nutritional status. Knowledge of AIDS was indicated by "Have you ever heard about AIDS (yes versus no)?" "A person can use a condom to avoid getting AIDS (yes versus no)" was used as an indicator of AIDS prevention knowledge. Antenatal practices by a pregnant woman who had given birth during the last 5 years preceding the BDHS survey were measured by using the number of antenatal visits. If the number of visits was less than 4, they were termed as "inadequate", otherwise termed as "adequate". The cut off point '4' was chosen arbitrarily. Although the period of 5 years is a subject to recall bias, all the three BDHS surveys that were conducted for women in 1993–94, 1996–97 and 1999–2000 had used such a period for this question. The composite variable of safe delivery practices was constructed by using three variables:

• Last delivery was assisted by a qualified doctor (yes = 1, no = 0)

• Last delivery was assisted by a nurse/midwife/paramedic (yes = 1, no = 0)

• Last delivery was assisted by a trained traditional birth attendant (TBA) (yes = 1, no = 0)

The total score for the composite variable of safe delivery varied from 0 to 3, where the score 0 indicated "not assisted by any skilled sources" and score 1 to 3 indicated "assisted by at least one skilled source". According to the data, 65%, 19.3% and 15.7% of the last deliveries were assisted by none, one and two skilled sources, respectively. None of the deliveries was assisted by the above-mentioned three sources, which seems logical. For instance, if any complication arises during delivery which cannot be handled by TBA (widely available), then TBA can seek assistance from a qualified medical doctor.

### Analysis

First simple analyses were performed to test the unadjusted differences between women living in slums compared to those not living in slums for many variables for five dependent variables. Multiple linear regression analysis was performed for BMI (continuous), whereas multiple logistic regression analyses were performed for dichotomous dependent variables. All the multiple regression analyses included four socio-economic variables namely childhood place of residence (countryside versus town/city), a composite variable of housing characteristics (poor versus not-poor), a composite variable of mass media access (not at all versus at least one media), and education (no education, primary education, secondary and higher education) in addition to the variable slum. Age was not considered in the regression model as it was adjusted at the time of matching slum (cases) and non-slum (controls) women. Regression coefficients and 95% confidence intervals including p-values were presented for BMI. For the other dichotomized dependent variables, odds ratios (OR) and corresponding 95% confidence intervals (CI) were presented.

## Results

Unadjusted differences between women living in slums compared to those not living in slums for the selected independent variables are presented in Table 1. The average age of the women was 30 years in both slum and non-slum areas (P = 0.969). Rate of illiteracy was significantly higher among women living in slums (41%) than those women living in non-slums (31%). Migration rate from country side was also significantly higher among slum (82%) than non-slum (69%) women. Husband's education was significantly lower for the women living in slums than those living in non-slums. Unskilled labor among the husbands of women living in slums was significantly more frequent than those husbands not living in slums. Six household characteristics and a composite variable of household characteristics by women living in slums and non-slums revealed that women in slums were significantly poorer than women in non-slums. For instance, 43% of women in slums had a modern toilet facility as compared to 69% in non-slums. The composite variable of household characteristics also differed significantly by women living in slums and non-slums. Mass media variables as well as the composite variable of mass media indicated that mass media access was significantly higher among women in non-slums than women in slums. For instance, reading the newspaper not at all was 85% among women in slums as compared to 72% among women in non-slums. Similarly watching TV not at all was 35% among women in slums whereas, this figure was 28% among women in non-slums.

Unadjusted results (Table 2) revealed that mean BMI was significantly lower among the women who: were living in a slum, lived in the countryside during childhood, had poorer household characteristics, had no access to media, and were not educated. In the multiple regression analysis, the significantly negative association of BMI with slum variable disappeared while all other associations of BMI with childhood place of residence, composite variable of household characteristics, composite variable of mass media access, and education remained significant. These results indicated that the difference of mean BMI by women living in slums and non-slums was confounded by other mentioned factors.

**Table 2:** BMI by selected socio-economic variables

Characteristics	Categories	Unadjusted comparison		Multiple linear regression		
			
		BMI N (mean ± SD)	P	Coefficient	95% CI (LL, UL)^#^	P
Slum	Non-slum	477 (21.4 ± 3.9)	0.002	Reference		
	Slum	118 (20.2 ± 3.5)		-0.33	-1.04, 0.39	0.369
Place of residence	Town/city	168 (22.5 ± 4.3)		Reference		
	Country side	427 (20.6 ± 3.5)	<0.001	-1.21	-1.85, -0.57	<0.001
Composite variable of household characteristics	Poor	167 (19.2 ± 2.6)		Reference		
	Not-poor	425 (21.9 ± 4.0)	<0.001	1.51	0.82, 2.20	<0.001
Composite variable of mass media access	Not at all	112 (19.2 ± 2.9)		Reference		
	At least one media	482 (21.6 ± 3.9)	<0.001	1.19	0.44, 1.94	0.002
Education	No education	197 (19.7 ± 3.2)		Reference		
	1–5 years education	171 (20.6 ± 3.4)	0.011	0.36	-0.37, 1.08	0.331
	6+ years education	227 (22.8 ± 4.0)	<0.001	1.94	1.21, 2.67	<0.001

^#^LL = Lower limit and UL = Upper limit

Associations of AIDS knowledge (indicated by ever heard about AIDS) with five selected variables are shown in Table 3. While the rate of ever had heard about AIDS was significantly lower among women in slums (69%) than those women living in non-slums (82%) in unadjusted analysis, this significance disappeared (P = 0.125) in multiple logistic regression analysis. Childhood place of residence, a composite variable of mass media access and education appeared as significant predictors of AIDS knowledge among women. Similar results were found for the variable "use of condom to avoid AIDS" (Table 4).

**Table 3:** AIDS knowledge by selected socio-economic variables

Characteristics		Unadjusted comparison		Multiple logistic regression		
			
		N (% yes^¶^)	P	OR	95% CI	P
Slum	Non-slum	480 (82.3)	0.001	1.00		
	Slum	120 (69.2)		0.70	0.40–1.12	0.125
Place of residence	Town/city	169 (95.3)		1.00		
	Country side	431 (73.5)	<0.001	0.20	0.09–0.43	<0.001
Composite variable of household characteristics	Poor	169 (63.9)		1.00		
	Not-poor	428 (85.7)	<0.001	1.51	0.93–2.46	0.099
Composite variable of mass media access	Not at all	114 (50.9)		1.00		
	At least one media	485 (86.4)	<0.001	3.46	2.13–5.63	<0.001
Education	No education	199 (62.8)		1.00		
	1–5 years education	173 (80.3)	<0.001	2.15	1.27–3.62	0.004
	6+ years education	228 (93.9)	<0.001	5.42	2.77–10.60	<0.001

^¶^Percentage of reporting ever heard about AIDS

**Table 4:** Condom use to avoid AIDS by selected socio-economic variables

Characteristics		Unadjusted comparison		Multiple logistic regression		
			
		N (% yes^¶^)	P	OR	95% CI	P
Slum	Non-slum	480 (35.6)		1.00		
	Slum	120 (25.8)	0.042	1.06	0.64–1.78	0.815
Place of residence	Town/city	169 (52.7)		1.00		
	Country side	431 (26.2)	<0.001	0.43	0.28–0.64	<0.001
Composite variable of household characteristics	Poor	169 (16.6)		1.00		
	Not-poor	428 (40.2)	<0.001	1.60	0.95–2.69	0.078
Composite variable of mass media access	Not at all	114 (6.1)		1.00		
	At least one media	485 (40.2)	<0.001	5.81	2.58–13.10	<0.001
Education	No education	199 (14.1)		1.00		
	1–5 years education	173 (30.6)	<0.001	2.25	1.30–3.88	0.004
	6+ years education	228 (53.1)	<0.001	4.55	2.70–7.69	<0.001

^¶^Percentage of reporting condom use as a method of AIDS prevention

Unadjusted associations of antenatal visits during the last pregnancy with the predictor variables (Table 5) showed that all the five variables were significantly associated with antenatal visits. However, in multiple logistic regression analyses 3 of these 5 associations became insignificant except for the variable of childhood place of residence and education. This means that the unadjusted significant differences of antenatal visits by women living in slums and non-slums were strongly influenced by childhood place of residence and education.

**Table 5:** Antenatal visits by selected socio-economic variables

Characteristics		Unadjusted comparison		Multiple logistic regression		
			
		N (% yes^¶^)	P	OR	95% CI	P
Slum	Non-slum	212 (30.7)		1.00		
	Slum	62 (17.7)	0.046	0.84	0.37–1.92	0.685
Place of residence	Town/city	60 (51.7)		1.00		
	Country side	214 (21.0)	<0.001	0.25	0.12–0.52	<0.001
Composite variable of household characteristics	Poor	84 (10.7)		1.00		
	Not-poor	190 (35.3)	<0.001	1.45	0.60–3.48	0.407
Composite variable of mass media access	Not at all	55 (12.7)		1.00		
	At least one media	219 (31.5)	<0.001	1.04	0.39–2.76	0.941
Education	No education	77 (6.5)		1.00		
	1–5 years education	81 (16.0)	0.059	2.44	0.79–7.61	0.123
	6+ years education	116 (50.0)	<0.001	12.11	4.08–35.93	<0.001

^¶^Percentage of adequate (4 or more) antenatal visits

Table 6 presents the information about the associations of safe delivery practices with the five selected predictors. While the unadjusted analysis seemed to result in a high significant difference (P = 0.001) between women living in slums and non-slums, this level became weaker (P = 0.038) in multiple logistic regression analysis. Only two variables namely childhood place of residence and education remained as strong predictors for safe delivery practices among women in Bangladesh.

**Table 6:** Safe delivery practices assisted by skilled sources by selected socio-economic variables

Characteristics		Unadjusted comparison		Multivariate		
			
		N (% yes^¶^)	P	OR	95% CI	P
Slum	Non-slum	212 (40.1)		1.00		
	Slum	62 (17.7)	0.001	0.45	0.21–0.96	0.038
Place of residence	Town/city	60 (60.0)		1.00		
	Country side	214 (28.0)	<0.001	0.30	0.15–0.58	<0.001
Composite variable of household characteristics	Poor	84 (19.0)		1.00		
	Not-poor	190 (42.1)	<0.001	1.28	0.62–2.65	0.501
Composite variable of mass media access	Not at all	55 (18.2)		1.00		
	At least one media	219 (39.3)	0.003	1.23	0.54–2.79	0.628
Education	No education	77 (16.9)		1.00		
	1–5 years education	81 (25.9)	0.169	1.54	0.67–3.55	0.309
	6+ years education	116 (53.4)	<0.001	4.34	1.93–9.78	<0.001

^¶^Percentage of safe delivery practices

## Discussion

According to the present study, education, childhood place of residence and access to mass media can play a significant role in reducing the gap between slum and non-slum women with respect to some public health variables in Bangladesh. In the context of Bangladesh, factors such as education, household characteristics, and access to mass media can be taken as indicators of poverty, because poverty causes high illiteracy, is associated with less access to mass media, and increases the likelihood of living in a poor household condition. Poverty also has the effect that people live in a poor environmental condition like a slum.

The finding of a lower BMI among women in slums compared to women living in non-slums in our study is consistent with the findings of other studies [10,11,32,33]. Widespread poverty and lower socio-economic conditions in slum areas are possible factors to explain the higher prevalence of malnutrition. For instance, under-nutrition was significantly associated with various factors such as higher age, unskilled labour, deficits in financial situation, overcrowding, household without electricity, household without tap water [10,33]. It is reported that 44% of the slum populations do not obtain the daily nutritional requirement [8].

Although Bangladesh has laid a solid foundation by recognizing that HIV/AIDS is a public health challenge, unfortunately low levels of HIV/AIDS knowledge still prevail in Bangladesh [34]. The present study showed that about 31% of the women in slums and 18% of women living not in slums did not even know the name of a disease called AIDS. This result is consistent with the findings of another study in India [35], which is the most affected country in terms of HIV/AIDS related burden of disease in South Asia and geographically very close to Bangladesh. Improving education, increasing access to the mass media such as television [36,37], street skits [35], family health awareness campaign, and involvement of community leaders [36] may be useful in enhancing the awareness among underprivileged groups like people living in slums. Concepts like 'each one teach ten' on a one-to-one basis or 'each one once-in-a-month' or 'my target-my area' may also be helpful in this regard [35].

HIV is transmitted mainly through unprotected sexual contacts. Condom use is the most effective method for protection against STDs including AIDS. One recent study reported that individuals infected with STDs were 5–10 times more likely than uninfected individuals to acquire or transmit HIV through sexual contact [38]. Although access to mass media such as radio, television, and newspaper can significantly increase the knowledge of protecting AIDS, many people especially in the slum areas still have no access to such media in Bangladesh. Therefore, much more efforts must be made to achieve a universal access to mass media. Increasing facilities for improved women education particularly in the slum areas may be another important strategy to improve the knowledge of AIDS prevention thorough condom use.

Antenatal services, which usually indicate pregnancy related care provided by a health provider [39], can reduce both morbidity and mortality through the detection and treatment of pregnancy-related illness and complications [40]. Particularly antenatal check ups and safe delivery practices assisted by skilled providers are extremely necessary for Bangladeshi women where the maternal mortality is higher as compared to other developing countries [41]. Other studies and the present study indicate that most of the women living in slums did not receive prenatal care [25] and did not seek any assistance from skilled providers for the last pregnancy complications [41], although a large proportion of women especially in urban slums experience serious delivery-related complications and/or postpartum morbidity [11,12]. Not only mothers, new born babies also receive benefits from these services. For instance, the incidence of low birth weight was 37% among mothers who had no antenatal check-up, while it was only 16% among those who had a check-up more than 7 times [23]. According to the multiple logistic regression analysis, the unadjusted discrepancies between women in slums and non-slums for antenatal visits and safe delivery practices provided by skilled sources can be explained by existing differences in education, childhood place of residence and access to mass media.

The study has several advantages. For example, it focused on several indicators related to public health; it used the data from all areas of Bangladesh; and compared women living in slums with those women not living slums for the first time. However, several limitations such as secondary data analysis and small sample size for women living in slums (related to possible selection bias and imprecise estimates) may limit the generalization of the findings. Particularly the women living in slums were very few in numbers whereas many people in Bangladesh are living in slums. We explored the question why slum households were underrepresented by BDHS by contacting a NIPORT authority (Ahmed Al-Sabir, Director, personal communication, 2008), the main organization in Bangladesh for BDHS 2004. According to his comment, BDHS did not have a special focus on people living in slums. The cross-sectional study design is another limitation. A period of 5 years for antenatal visits during the last pregnancy may also be subject to recall bias.

In conclusion, unadjusted differences between women living in slums and non-slums for several public health-related variables namely BMI, ever heard about AIDS, use of condom to avoid AIDS, and antenatal visits are misleading, because the inclusion of several socio-economic factors namely education, a composite variable of mass media and childhood place of residence make such differences insignificant and thus elucidate these differences as spurious. These results indicate that improving education for women, especially for those who are living in slums, can minimize the existing differences for public health outcomes. Increasing access to mass media has also potentials for effectively improving disease prevention and health promotion. Our findings suggests that associations with childhood place of residence, mass media access and public health education should be considered before making any inference based on slum versus non-slum comparison. Further studies, based on large sample sizes from both slum areas and non-slum areas, are necessary to validate findings.

## Competing interests

The authors declare that they have no competing interests.

## Authors' contributions

MMHK conceived the idea and performed the statistical analysis. AK assisted in drafting the manuscript and interpreting the results. Both authors read and approved the final manuscript.

## Pre-publication history

The pre-publication history for this paper can be accessed here:



## References

[B1] World Urban Forum (2004). Dialogue on the urban poor: improving the lives of slum-dwellers. HSP/WUF/2/6.

[B2] Riley LW, Ko AI, Unger A, Reis MG (2007). Slum health: Diseases of neglected populations. BMC Int Health Human Rights.

[B3] Sclar ED, Northridge ME (2003). Slums, slum dwellers, and health. Am J Public Health.

[B4] Centre for Urban Studies (CUS), National Institute of Population Research and Training (NIPORT) and Measure Evaluation (2006). Slums of Urban Bangladesh: Mapping and Census, 2005.

[B5] Sclar ED, Garau P, Carolini G (2005). The 21^st ^century health challenge of slums and cities. Lancet.

[B6] Harpham T (1986). Health and the urban poor. Health Policy Plann.

[B7] Huq-hussain S (1996). Female migrants in an urban setting – the dimensions of spatial/physical adaptation. The case of Dhaka. Habitat Int.

[B8] Huq-hussain S (1995). Fighting poverty: the economic adjustment of female migrants in Dhaka. Environ Urban.

[B9] Hussain A, Ali SM, Kvale G (1999). Determinants of mortality among children in the urban slums of Dhaka city, Bangladesh. Trop Med Int Health.

[B10] Pryer JA, Rogers S, Rahman A (2003). Factors affecting nutritional status in female adults in Dhaka slums, Bangladesh. Soc Biol.

[B11] Uzma A, Underwood P, Atkinson D, Thackrah R (1999). Postpartum health in a Dhaka slum. Soc Sci Med.

[B12] Fronczak N, Antelman G, Moran AC, Caulfield LE, Baqui AH (2005). Delivery-related complications and early postpartum morbidity in Dhaka, Bangladesh. Int J Gynaecol Obstet.

[B13] Rahim MA, Vaaler S, Keramat Ali SM, Khan AK, Hussain A, Nahar Q (2004). Prevalence of type 2 diabetes in urban slums of Dhaka, Bangladesh. Bangladesh Med Res Counc Bull.

[B14] Rahman S, Banu S, Nessa F (1989). Health situation of slum dwellers of metropolitan area of Dhaka. Bangladesh Med Res Counc Bull.

[B15] Haq SA, Darmawan J, Islam MN, Uddin MZ, Das BB, Rahman F, Chowdhury MA, Alam MN, Mahmud TA, Chowdhury MR, Tahir M (2005). Prevalence of rheumatic diseases and associated outcomes in rural and urban communities in Bangladesh: a COPCORD study. J Rheumatol.

[B16] Sabin KM, Rahman M, Hawkes S, Ahsan K, Begum L, Black RE, Baqui AH (2003). Sexually transmitted infections prevalence rates in slum communities of Dhaka, Bangladesh. Int J STD AIDS.

[B17] Ahsan H, Underwood P, Atkinson D (1998). Smoking among male teenagers in Dhaka, Bangladesh. Prev Med.

[B18] Rahman MM, Shahidullah M (2001). Risk factors for acute respiratory infections among the slum infants of Dhaka city. Bangladesh Med Res Counc Bull.

[B19] Chowdhury AM, Bhuiya A, Mahmud S, Abdus Salam AK, Karim F (2003). Immunization divide: who do get vaccinated in Bangladesh?. J Health Popul Nutr.

[B20] Pryer JA, Rogers S, Rahman A (2004). The epidemiology of good nutritional status among children from a population with a high prevalence of malnutrition. Public Health Nutr.

[B21] Arifeen SE, Black RE, Caulfield LE, Antelman G, Baqui AH, Nahar Q, Alamgir S, Mahmud H (2000). Infant growth patterns in the slums of Dhaka in relation to birth weight, intrauterine growth retardation, and prematurity. Am J Clin Nutr.

[B22] Ahmed F (1992). Nutritional situation of Dhaka. Southeast Asian J Trop Med Public Health.

[B23] Nahar N, Afroza S, Hossain M (1998). Incidence of low birth weight in three selected communities of Bangladesh. Bangladesh Med Res Counc Bull.

[B24] Arifeen S, Black RE, Antelman G, Baqui A, Caulfield L, Becker S (2001). Exclusive breastfeeding reduces acute respiratory infection and diarrhoea deaths among infants in Dhaka slums. Pediatrics.

[B25] Hoque A, Selwyn BJ (1996). Birth practice patterns in urban slums of Dhaka, Bangladesh. Women Health.

[B26] Mullick MS, Goodman R (2005). The prevalence of psychiatric disorders among 5–10 year olds in rural, urban and slum areas in Bangladesh: an exploratory study. Soc Psychiatry Psychiatr Epidemiol.

[B27] Ehrenberg JP, Ault SK (2005). Neglected diseases of neglected populations: thinking to reshape the determinants of health in Latin America and the Caribbean. BMC Public Health.

[B28] World Bank (2006). Bangladesh: country environmental analysis. Bangladesh Development Series Paper No 12.

[B29] Perry H, Weierbach R, Hossain I, Islam R (1998). Childhood immunization coverage in zone 3 of Dhaka City: the challenge of reaching impoverished households in urban Bangladesh. Bull World Health Organ.

[B30] Izutsu T, Tsutsumi A, Islam AM, Kato S, Wakai S, Kurita H (2006). Mental health, quality of life, and nutritional status of adolescents in Dhaka, Bangladesh: comparison between an urban slum and a non-slum area. Soc Sci Med.

[B31] National Institute of Population Research and Training (NIPORT), Mitra and Associates, and ORC Macro (2005). Bangladesh Demographic and Health Survey 2004.

[B32] Baqui AH, Arifeen SE, Amin S, Black RE (1994). Levels and correlates of maternal nutritional status in urban Bangladesh. Eur J Clin Nutr.

[B33] Pryer JA, Rogers S (2006). Epidemiology of undernutrition in adults in Dhaka slum households, Bangladesh. Eur J Clin Nutr.

[B34] NSAP (2004). Bangladesh country profile on HIV and AIDS 2004.

[B35] Kalasagar M, Sivapathasundharam B, Einstein TBA (2006). AIDS awareness in an Indian metropolitan slum dweller: a KAP (Knowledge, Attitude, Practice) study. Ind J Dent Res.

[B36] Bhatia V, Swami HM, Kaur AP (2004). An intervention study to enhance AIDS awareness among underprivileged population in Chandigarh. Indian J Dermatol Venereol Leprol.

[B37] Khan MMH, Kabir M, Mori M (2004). Impact of various sources of AIDS information among ever married men and women in Bangladesh. J Health Popul Dev Ctries.

[B38] Da Ros CT, Schmitt Cda S (2008). Global epidemiology of sexually transmitted diseases. Asian J Androl.

[B39] Matthews Z, Mahendra S, Kilaru A, Ganapathy S (2001). Antenatal care, care seeking and morbidity in rural Karnataka, India: results of a prospective study. Asia-Pacific Popul J.

[B40] Carroli G, Rooney C, Villar J (2001). How effective is antenatal care in preventing maternal mortality and serious morbidity? An overview of the evidence. Paediatr Perinat Epidemiol.

[B41] Khan MMH, Kabir M, Mori M (2005). Do various sources of disseminating AIDS information make significant difference to antenatal care in Bangladesh. J Health Popul Dev Ctries.

